# A severed IP ligament as a cause for trans-vaginal uterine bleeding post termination of pregnancy: a case report

**DOI:** 10.11604/pamj.2017.27.95.12435

**Published:** 2017-06-07

**Authors:** Madeeha Dean, Nicole Stamatopoulos, Thierry Vancaillie

**Affiliations:** 1 Royal Hospital for Women, Department of Obstetrics and Gynaecology, Randwick, Australia

**Keywords:** Gynaecology, dilatation, curettage, pregnancy, surgery

## Abstract

Uterine perforation is an uncommon yet serious complication of surgical management of first and second trimester termination of pregnancies. The rate of uterine perforation is under reported, as patients are usually asymptomatic. Although uncommon, uterine perforation can cause life-threatening complications for some patients. This case report discusses a second trimester surgical termination resulting in uterine perforation and haemorrhage secondary to an avulsion of the infundibulopelvic ligament and prolapse of the left fallopian tube and ovary into the uterine cavity. A literature search was undertaken to compare this case report to those previously published. To the best of our knowledge, this is the first case report in Australia that discusses a unique case of a severed infundibulo-pelvic ligament as a cause for trans-vaginal uterine bleeding post second trimester termination of pregnancy.

## Introduction

Surgical intervention (dilatation and curettage) for first and second trimester termination of pregnancy is quite common. Uterine perforation is an uncommon, yet serious complication of surgical termination of pregnancy [1]. Uterine perforations are usually not detected because most patients are asymptomatic [1]. The centre for disease control in the United States estimated that in 2008 the pregnancy termination rate was 16 per 1000 women between 15-44 years of age [2] and second trimester termination rate was less than 1 percent [3, 4]. The rate of uterine perforation due to surgical termination is 0.09-2.8% per 1000 cases [3]. This risk is increased with uterine anomalies and previous gynaecological surgeries [5–9]. While rare, this complication can be life threatening due to haemorrhage [5]. Other rare complications that have been reported in the literature include herniation of small bowel, omentum, appendix and fallopian tubes as a consequence of uterine perforation [1, 6]. The common symptoms reported in cases where fallopian tube herniation occurred in the uterine cavity are abdominal or pelvic pain, offensive vaginal discharge, mild vaginal bleeding, secondary infertility post surgical termination and abdominal distension [1, 3, 5, 6, 10–13]. There is support around the use of ultrasound guidance during surgical termination to decrease procedure related morbidity [5, 7]. The use of proper imaging techniques-transvaginal ultrasound and MRI allows for prompt diagnosis and early intervention. The ultrasonographic finding of a fallopian tube incarceration is a hyperechoic tubular structure in the myometrium [1]. Fallopian tube herniation is often misdiagnosed as an endometrial polyp or submucous myoma on transvaginal ultrasound, saline infusion sonography and hysterosalpinography [3, 7, 8, 11–13]. After imaging if there is any indication of an incarcerated intra-abdominal or pelvic structure, surgical treatment is recommended1. Most commonly, hysteroscopy, laparoscopy and salpingectomy or salpingoplasty are used, depending on anatomy and patient pregnancy wishes [1, 6]. In some situations or where there is lack of surgical expertise, a laparotomy may be necessary [10]. Post-operatively, a hysterosalingography and hysteroscopy 3 months post procedure to assess tubal patency, uterine wall repair and synechiae is recommended [1] as well as the discussion regarding contraception in the case of an unwanted pregnancy.

## Patient and observation

A 31 year old woman was transferred by ambulance from a private clinic following an elective surgical termination of pregnancy (TOP) at 19 weeks gestation with a postpartum haemorrhage (PPH). Post procedure the patient received several oxytocics including: several doses of syntocinon, a syntocinon infusion, 750 micrograms of intramuscular ergometrine in 3 doses as well as 400 micrograms of rectal misoprostol. Estimated blood loss at the clinic was estimated to be 1000ml. On arrival at the emergency department, the patient was transferred to the resuscitation bay. She was alert, orientated and responsive, but diaphoretic. Her airway was patent. She had a heart rate of 80 and a blood pressure of 115/80. When the gynaecology registrar arrived, the patient had 2 large bore cannulas in, intravenous (IV) fluids and a Syntocinon infusion running. There were multiple attempts to take bloods for a full blood count (FBC), group and hold (G & H) and coagulation profile with no success. A brief history was taken. This was a second trimester elective TOP following a normal morphology ultrasound. It is unknown if the TOP was under ultrasound guidance. The patient had a BMI of 35, had five previous pregnancies; two children via normal vaginal delivery and two previous surgical TOPs (G5 P2 T2). She had no previous history of PPH and delivered at term. The patient was not on any medication and had an allergy to penicillin, which gave her a rash. She is a non-smoker and was otherwise well. On examination she was haemodynamically stable with a fundus below the umbilicus and no abdominal discomfort. External genital exam showed a large blood clot overlying the perineum. Bimanual exam expressed many clots from the uterus. A quick bedside ultrasound confirmed clots inside the uterus. The patient was immediately transferred to the operating theatre for an examination under anaesthetic (EUA). Prior to being anaesthetised, an FBC, G & H and coagulation profile were taken and urgently sent by the anaesthetic staff. Starting haemoglobin (Hb) was 118. The patient was anaesthetised and put into lithotomy position, prepped and draped. An indwelling catheter was inserted and a manual examination of the endometrial cavity was commenced.

Approximately another 200ml of clot was evacuated from the endometrial cavity. On repeat examination, there was suspicion of a left sided uterine perforation when a small amount of what appeared to be omentum was removed. On further examination, a mass was felt in the right lower quadrant of the uterus. It was suspected that there were still fetal parts left in the cavity. The consultant was called. No fetal parts were seen with ultrasound. What was thought to be fetal parts was actually an ovary. A specialist gynaecologist was called into the operating theatre. The decision was made to perform a laparoscopy as the uterus was approximately 14-16 weeks size at this stage. The findings on laparoscopy included several hundred milliliters of blood in the pelvis that had pooled at the left lateral uterine wall. A left lateral wall perforation of the uterus could also be seen. The left ureter and uterine artery appeared to be intact. The left ovary was partially pulled into the uterine defect and was extracted back into the pelvic cavity with a grasper. No obvious source of bleeding could be found initially-just a general ooze surrounding the perforation. Surgicel was used to assist with haemostasis. Further inspection of the upper pelvis showed an avulsed left infundibulo-pelvic (IP) ligament as the cause of the bleeding in the pelvis, which was surprisingly minimal. It appeared that during the TOP, the instrument used to perform the procedure had perforated the left lateral uterine wall, missed both the uterine artery and ureter, grasped the left ovary and avulsed the left infundibulo-pelvic ligament. The left ovary was pulled into the uterus and subsequently ongoing uterine bleeding occurred from the ovarian side of the IP ligament. Bipolar diathermy was used on both ends of the IP ligament to ensure complete haemostasis. It was estimated that the patient had lost 2000ml of blood to this point. During the laparoscopy, a massive transfusion protocol was activated. The patient was given cryoprecipitate and fresh frozen plasma intraoperatively and a total of 4 units of packed red cells during the course of her admission. She was admitted to the acute care unit post operatively for close monitoring. The patient and her partner were debriefed on the findings. She recovered quickly, considering the serious nature of this complication and was discharged on day 2 postoperatively. She was asymptomatic despite an Hb of 80 and was given Ferrinject prior to discharge. She also declined contraception. The patient returned 2 weeks later for follow up. She was completely well at review. She still declined contraception and advised her partner was taking responsibility for this. The complication was also discussed with the clinic and the doctor who performed the procedure.

## Discussion

To the best of our knowledge, this is the first case report in Australia that discusses a second trimester surgical termination resulting in uterine perforation and haemorrhage secondary to an avulsion of the infundibulopelvic ligament and prolapse of the left fallopian tube and ovary into the uterine cavity. A literature review was completed on PubMed, CINAHL, EMBASE, MEDLINE and Google Scholar to find articles similar to this case. The literature review resulted in 13 articles that were somewhat applicable to the current case. However, two articles were excluded as the full-text were in foreign language [14, 15]. Most case reports discussed fallopian tube incarceration detected either in the short term or up to 5 years post termination of pregnancy in the first trimester [5–10, 12]. Kondo et al discussed uterine perforation post dilatation and curettage for retained products of conception [1, 12, 13]. Chung and Cheung discussed a case of first trimester elective surgical termination in a patient with a previous LSCS that resulted in an avulsed fallopian tube secondary to a uterine perforation [9]. Alanbay et al reported a case that investigated a female for secondary infertility post first trimester dilatation and curettage. Diagnostic laparoscopy showed that the right fallopian tube and distal part of the infundibulopelvic ligament were adherent to the posterior fundal uterine wall but the patient's clinical parameters are different to ours [8].

## Conclusion

This unique case report raises awareness about uterine perforation secondary to surgical termination of pregnancies. It demonstrates the management of a severed infundibulo-pelvic ligament as a cause for trans-vaginal uterine bleeding post termination of pregnancy in the second trimester.

## Competing interests

The authors declare no competing interest.
